# Tapered discontinuation vs. maintenance therapy of antipsychotic medication in patients with first-episode schizophrenia: Obstacles, findings, and lessons learned in the terminated randomized clinical trial TAILOR

**DOI:** 10.3389/fpsyt.2022.910703

**Published:** 2022-07-22

**Authors:** Anne Emilie Stürup, Carsten Hjorthøj, Nikolai Albert, Signe Dolmer, Merete Birk, Bjørn H. Ebdrup, Lene Falgaard Eplov, Heidi Jensen, Ditte Lammers Vernal, Helene Speyer, Ole Mors, Merete Nordentoft

**Affiliations:** ^1^Copenhagen Research Center for Mental Health (CORE), Mental Health Center Copenhagen, Copenhagen University Hospital, Copenhagen, Denmark; ^2^Section of Epidemiology, Department of Public Health, University of Copenhagen, Copenhagen, Denmark; ^3^Psychiatry Øst, Region Sjælland, Roskilde, Denmark; ^4^Psychosis Research Unit, Department of Clinical Medicine, Aarhus University Hospital, Aarhus, Denmark; ^5^Center for Neuropsychiatric Schizophrenia Research (CNSR), Mental Health Centre Glostrup, University of Copenhagen, Glostrup, Denmark; ^6^Department of Clinical Medicine, Faculty of Health and Medical Sciences, University of Copenhagen, Copenhagen, Denmark; ^7^Unit for Psychiatric Research, Psychiatry, Department of Clinical Medicine, Aalborg University Hospital, Aalborg University, Aalborg, Denmark

**Keywords:** first-episode schizophrenia, discontinuation, tapering, maintenance, antipsychotics, remission, relapse, design

## Abstract

**Aim:**

Evidence is insufficient regarding the consequences of discontinuing vs. maintaining antipsychotic medication in patients with first-episode schizophrenia. Our aim was to examine tapered discontinuation vs. maintenance treatment regarding remission of psychotic symptoms and impact on other areas.

**Methods:**

Patients included had a diagnosis of schizophrenia, were treated with antipsychotic medication, and were in remission of psychotic symptoms. Participants were randomized to tapered discontinuation or maintenance treatment with antipsychotic medication. Assessments were undertaken at baseline and after 1-year. The primary outcome was remission of psychotic symptoms without antipsychotic medication.

**Results:**

The trial was terminated due to insufficient recruitment. In total, 29 participants were included: 14 in the tapering/discontinuation group and 15 in the maintenance group. Adherence to maintenance treatment was poor. At 1-year follow-up, remission of psychotic symptoms without antipsychotic medication for 3 months was observed in five participants in the tapering/discontinuation group and two in the maintenance group.

**Conclusion:**

Due to insufficient recruitment this study does not provide a conclusion on whether unfavorable outcomes or advantages follow tapering of antipsychotic medication. Recruitment and adherence to maintenance treatment encountered obstacles. Based on experiences from this trial, we discussed alternative study designs as consistent evidence is still needed on whether to continue or discontinue antipsychotic medication in remitted patients with first-episode schizophrenia.

**Clinical trial registration:**

https://www.clinicaltrialsregister.eu/ctr-search/trial/2016-000565-23/DK, EU Clinical Trials Register—EudraCT no. 2016–000565–23.

## Introduction

Patients with first-episode schizophrenia are recommended treatment with antipsychotic medication to reduce psychotic symptoms (1–3), and growing evidence show antipsychotics may prevent relapse (4, 5). However, the question remains, what is the optimal duration of antipsychotic medication to prevent relapse after remission of psychotic symptoms as some patients experience no relapse after discontinued antipsychotic medication (6, 7). On the one hand, discontinuing antipsychotic medication might increase the risk of relapse of psychotic symptoms (8) and decrease social function (9). On the other hand, continued antipsychotic medication might be followed by serious side effects, e.g., movement disorders, sedation, obesity, diabetes and cardiovascular disease (10). Furthermore, discontinuation of antipsychotic medication has previously been linked to improved neurocognitive function (11). Thus, evidence-based recommendations are crucial when prescribing antipsychotic medication as well as the proper time for discontinuation of antipsychotics in dialogue with patients and relatives.

Despite the increased risk of relapse, follow-up studies of first-episode psychosis, including first-episode schizophrenia, show that approximately fifty percent of patients achieve remission from psychotic symptoms after 10 years (12, 13) and almost half have discontinued antipsychotic medication (6, 13). Furthermore, a randomized clinical trial comparing dose reduction to maintenance treatment found that patients who initially received the intervention of dose reduction had a higher chance of achieving functional remission 5 years after the intervention than patients initially receiving maintenance treatment (14). A recent systematic review of randomized controlled trials reported that patients with first-episode psychosis in remission of psychotic symptoms had a higher risk of relapse when discontinuing antipsychotic medication (53%) compared to maintenance treatment (19%). Although, only a few studies were conducted in early intervention services, and in the majority placebo was used in the discontinuation intervention (8).

We designed a randomized clinical trial to investigate the feasibility of tapering/discontinuing antipsychotic medication in first-episode schizophrenia. Due to insufficient recruitment, we are unable to answer this question in this paper. The original design of the trial is reported in the study protocol (15). While this paper reports the outcome of the participants included, it also describes the lessons learned and how further studies could learn from the obstacles encountered.

## Methods

### Trial design and approvals

The TAILOR study was an investigator-initiated, multicenter, randomized, assessor-blinded, parallel-group, superiority designed clinical trial. The design of the study was described in the study protocol (15). Sample size calculation was done for the primary outcome and several of the secondary outcomes (15). A study sample of 250 patients (125 participants in each treatment arm) was estimated to be realistic and yield a clinically relevant difference of the primary outcome.

The study obtained approvals from the Regional Research Ethical Committee in the Capital Region of Denmark (no 53322), the Medical Committee (no 2016011815), and the Danish Data Agency (RHP-2017-011, I-Suite no 05437). Furthermore, the trial was registered with EudraCT no 2016-000565-23. The trial was monitored by GCP (Good Clinical Practice) (16). Written informed consent was obtained after written and oral communication between researcher and participant.

Important changes not described in the study protocol was the addition of one more trial site, change of inclusion criteria from patients having received 11 months of treatment in a specialized early intervention team (in Denmark named OPUS) to having 12 months left of treatment in an OPUS team and use of a mobile application for reporting warning signs of psychosis was aborted. Finally, 2 and 5-year follow-ups were canceled.

The trial ended prematurely because of recruitment problems which will be elaborated on in the discussion section.

### Participants and randomization

Study participants were referred by their physician in the early intervention services (OPUS), and eligibility was confirmed by the researcher at the baseline interview.

The inclusion criteria were: (1) First treatment in OPUS with the diagnosis schizophrenia (ICD10 DF20, except DF20.6) or persistent delusional disorder (DF22). (2) Minimum 3 months remission of psychotic symptoms [Scale for the Assessment of Positive Symptoms (SAPS) global scores ≤ 2] (17) and within the first 11 months of treatment in OPUS. (3) In treatment with antipsychotic medication (oral or long-acting injection). (4) Minimum age 18 years. (5) Fluent in Danish. (6) Informed consent.

The exclusion criteria were: (1) Patient in forensic psychiatry. (2) Treatment with clozapine. (3) Pregnancy or breastfeeding. (4) Previous admission to a psychiatric hospital due to a psychotic relapse while treated with antipsychotic medication or tapering of antipsychotic medication.

The withdrawal criteria were: If the patient within the year of intervention met exclusion criteria 1, 2, or 3, were no longer an out-patient in OPUS, or withdrew informed consent. In case of withdrawal from the intervention arm, the participants were asked to participate in the follow-up interviews.

The randomization is described in the study protocol.

### Intervention

Participants were randomized to 1 year of either tapered discontinuation of antipsychotic medication (tapering/discontinuation group) or treatment as usual with maintenance therapy with antipsychotic medication (maintenance group). Both groups received usual non-pharmacological treatment in their OPUS team during the intervention year comprising assertive community treatment including psychoeducation, family involvement, and social skills training. The interventions were carried out by the participants' psychiatrists in the OPUS teams. Safety of the participants was ensured by regular contacts with the OPUS teams, monthly assessment by phone with the researchers evaluating psychotic symptoms (SAPS phone), and monitoring by Good Clinical Practice (GCP), including reports of serious adverse events and serious adverse reactions.

#### Tapered discontinuation

The psychiatrist in OPUS managed the tapering of antipsychotic medication, and guidelines were: (1) 25% monthly reduction of baseline dose, (2) with a minimum of five half-lives between each reduction, (3) duration of at least 6 months, and (4) with regular assessments and evaluations. Tapering was paused for 3 months when the minimum effective dose (18) was reached to ensure stabilization before finalizing the tapering over ~3 months. If the participant experienced psychotic deterioration, psychotic relapse, or individual warning signs evaluated by the clinicians as signs of deterioration, it was recommended to increase the antipsychotic dose. Tapering could be resumed after 3 months' remission of psychotic symptoms. Despite deterioration or relapse, the participant stayed in the tapering/discontinuation group. The intervention of tapering was monitored by GCP to ensure safety, and that tapering followed protocol.

#### Maintenance treatment

Maintenance therapy was regarded as treatment as usual with antipsychotic medication. A switch of antipsychotics and 25% dose adjustments were allowed. Tapering or discontinuation due to effect, side effects, or participant's wish was accepted. The clinician encouraged the participant to continue taking prophylactic antipsychotics if this was still considered a safe option. However, if the participant insisted on discontinuing antipsychotics, the clinician helped the participant in this process to ensure safety.

### Assessment and outcomes

Baseline and 1-year follow-up assessments were conducted at the research units or the OPUS teams and were carried out by medical doctors or psychiatric nurses, who were certified and trained as interviewers and assessors. To assess psychotic relapse, study participants were assessed by monthly phone interviews. The primary outcome measure was remission of psychotic symptoms (SAPS ≤ 2 in all global scores in minimum 3 months) without taking antipsychotic medication during the past 3 months at 1-year follow-up. Secondary outcomes, exploratory outcomes, and safety measures were assessed as planned at the 1-year follow-up. The outcome measures for 2 and 5-year follow-ups e.g., recovery and repetition of first-year follow-up outcomes were all dismissed since the trial ended prematurely. The complete list of planned outcomes assessed was described in the protocol (15).

### Statistical methods

The original statistical plan was described in the protocol, including sample size calculations. However, due to the early termination of the trial, we do not report statistical analyses or *p*-values since it could potentially be misleading (19). Missing single construct values of variables were not accepted except when stated otherwise in instructions for the instrument. An outcome was not included if the outcome was missing for more than 40% of participants.

## Results

### Recruitment and inclusion

**Figure 1:**
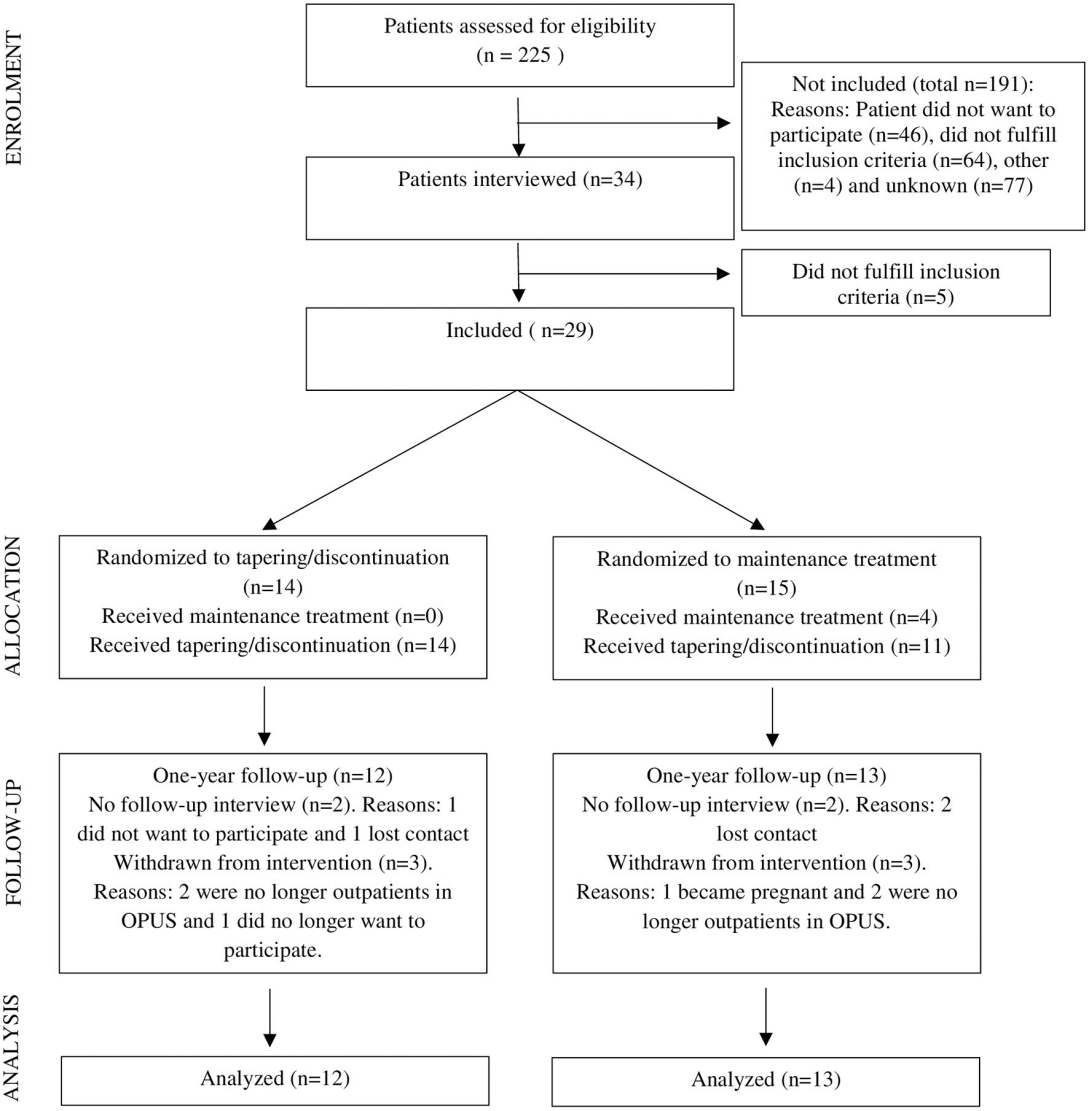
Flow diagram of enrolment, allocation, 1-year follow-up, and analysis (adapted figure from the CONSORT template licensed under CC BY 2.0).

The TAILOR trial started recruiting in April 2017 and stopped in January 2019. The trial was terminated in January 2020 with the conclusion of the 1-year follow-up interview of the last included participant. In total, 225 patients were referred to the study. The participant flow is illustrated in Figure 1. Eligibility was assessed in 225 patients; 191 patients did not meet inclusion criteria in the first screening, including 46 who did not want to participate. Thus, 34 patients were interviewed for inclusion, and hereof five patients did not meet the study criteria. In total, 29 participants were included and randomized during the 20 months the trial was actively recruiting.

During the study, five patients were withdrawn from the randomized treatment, two of whom participated in the 1-year follow-up interview (one from each treatment arm). Two participants in the tapering/discontinuation group and two in the maintenance group were lost to follow-up. One patient lost to follow-up in the tapering/discontinuation group had a psychotic relapse. At the 1-year follow-up, 25 participants were interviewed. Information from monthly telephonic SAPS interviews and antipsychotic medication was available for all 29 included participants.

Patients and clinicians explained that the lack of recruitment was due to preferences of treatment and hence non-acceptance of randomly being assigned to another treatment. The fear of relapse and adverse events when discontinuing antipsychotics was most pronounced among clinicians, and the readiness to discontinue antipsychotics was most pronounced in patients. Furthermore, clinicians reported that tapering antipsychotics was already implemented early in the illness course, and the timeframe was too narrow for the patient to obtain remission and receive a 1-year intervention during the 2-year treatment in the OPUS team.

### Baseline characteristics

Included participants represented a highly selected group, and the sociodemographic and clinical characteristics are presented in Table 1. Participants were young, the majority lived independently, and only a few had competitive employment. All participants except one (persistent delusional disorder) were diagnosed with schizophrenia. In the tapering/discontinuation group, the median duration of untreated psychosis (DUP) was 81 months (range 1.0–306.0 months), and in the maintenance group, the median was seven months (range 1.0–252.0 months). The study population had low levels of psychotic and negative symptoms, moderate functional difficulties, and a low degree of side effects. Except for DUP, the randomization resulted in two seemingly similar groups despite the modest number of participants.

**Table 1:** Sociodemographic and clinical characteristics of patients with first-episode schizophrenia included at baseline.

	**Tapering/discontinuation group (*****n*** = **14)**	**Maintenance group (*****n*** = **15)**	**Total (*****n*** = **29)**
	**Mean (SD)**	**Mean (SD)**	**Mean (SD)**
Age (years)	24.9 (6.8)	25.3 (7.9)	25.1 (7.2)
	Count (column %)	Count (column %)	Count (column %)
Gender (female)	9 (64.3)	8 (53.3)	17 (58.6)
Married or cohabiting	2 (14.3)	1 (6.7)	3 (10.3)
Children	2 (14.3)	2 (13.3)	4 (13.8)
Completed lower secondary school or less	7 (50.0)	5 (33.3)	12 (41.4)
Student	1 (7.1)	5 (33.3)	6 (20.7)
Welfare payment	11 (78.6)	8 (53.3)	19 (65.5)
**Living arrangement**			
Living independently	9 (64.3)	13 (86.7)	22 (75.9)
Living with parent(s)	3 (21.4)	2 (13.3)	5 (17.2)
Supported housing	2 (14.3)	0 (0.0)	2 (6.9)
Ordinary work	1 (7.1)	0 (0.0)	1 (3.4)
**Diagnosis of psychosis**			
Schizophrenia	14 (100)	14 (93.3)	28 (96.6)
Delusional disorder	0 (0.0)	1 (6.7)	1 (3.4)
Diagnosis of psychoactive substance use or dependence	2 (14.3)	3 (20.0)	5 (17.2)
	Median (range)	Median (range)	Median (range)
Duration of untreated psychosis in months	81.0 (1.0–306.0)	7.0 (1.0–252.0)	26.0 (1.0–306.0)
	Mean (SD)	Mean (SD)	Mean (SD)
**Psychotic and negative symptoms past 3 months**			
Psychotic dimension (SAPS)	0.4 (0.6)	0.3 (0.6)	0.4 (0.6)
Negative dimension (SANS)	2.0 (0.9)	1.7 (0.7)	1.8 (0.8)
Disorganized dimension (SAPS)	0.3 (0.6)	0.1 (0.3)	0.2 (0.4)
**Medication side effects (UKU)**			
Psychic side effects^a^	7.6 (4.0)	4.0 (3.3)	5.8 (4.0)
Neurologic side	1.0 (1.4)	1.1 (1.4)	1.1 (1.4)
Autonomic side effects	1.6 (1.6)	1.9 (2.2)	1.8 (1.9)
Other side effects^b^	4.2 (4.1)	4.0 (3.8)	4.1 (3.8)
Patient score of interference	1.1 (0.9)	1.1 (0.6)	1.1 (0.8)
Rater score of interference	1.1 (0.9)	1.1 (0.5)	1.1 (0.7)
**Patient experience and quality of life**			
Quality of life (WHO-5 Wellbeing Index)	52.9 (19.1)	66.1 (14.5)	59.7 (17.9)
Service user's experience of support (INSPIRE S)^a^	76.3 (17.1)	73.9 (13.8)	75.1 (15.3)
Service user's experience of relationship (INSPIRE R)	93.6 (12.0)	83.7 (22.3)	88.6 (18.3)
Self-belief of coping (GSES mean)^a^	2.5 (0.6)	2.7 (0.6)	2.6 (0.6)
**Function**			
Cognitive function (BACS)	−2.1 (1.6)	−2.5 (1.1)	−2.3 (1.3)
Social function (PSP total score)	54.4 (8.3)	59.4 (10.1)	57.0 (9.4)
Level of functioning (GAF-F)	53.8 (7.7)	58.93 (9.8)	56.4 (9.1)
Social function (GSDS score except parental role)	7.5 (3.8)	6.3 (3.9)	6.9 (3.8)
	Count (column %)	Count (column %)	Count (column %)
Social dysfunction (GSDS cut off)	12 (85.7)	10 (66.7)	22 (75.9)
Sexual dysfunction (CSFQ-14 cut off)^c^	6 (54.5)	6 (40.0)	12 (46.2)
**Suicidality**			
Suicidal thoughts the past year (yes)	8 (57.1)	6 (40.0)	14 (48.3)
Suicide attempt the past year (yes)	3 (21.4)	2 (13.3)	5 (17.2)

*EQ-5D-3L (European Quality of Life – 5 Dimensions) was introduced during the study and therefor missing in the first 16 patients (>40% missing) and therefore not reported at baseline*.

a *Missing in one patient in the maintenance group*,

b *Missing in one patient in the Tapering/discontinuation group and three patients in the maintenance group*,

c *Missing in three patients in the Tapering/discontinuation group*.

*BACS, Brief Assessment of Cognition in Schizophrenia; CSFQ-14, Changes in Sexual Functioning Questionnaire; GAF-F, Global Assessment of Function; GSDS, Groningen Social Disabilities Schedule; GSES, General Self Efficacy; PSP, Personal and Social Performance Scale; SANS, Schedule for Assessment of Negative Symptoms; SAPS, Scale for the Assessment of Positive Symptoms; UKU, Udvalg for Kliniske Undersøgelser*.

### Adherence to treatment arm and antipsychotic medication

In the tapering/discontinuation group, 11 out of 14 participants discontinued their antipsychotic medication, and the remaining three were tapered. Seven of these 11 participants sustained discontinuation. In the maintenance group, four of 15 patients maintained their antipsychotic dosage, five participants discontinued their antipsychotics, and six patients tapered their antipsychotic medication. Four participants in the maintenance group sustained discontinuation at the 1-year follow-up.

Antipsychotic medication at baseline and 1-year follow-up are described in Table 2. At baseline, the mean dose of antipsychotic medication was 7.8 mg (range 2.4–16.0) haloperidol equivalents. At 1-year follow-up the mean dose antipsychotic was 3.8 mg haloperidol equivalents in the tapering/discontinuation group and 5.7 mg haloperidol equivalents in the maintenance group. The mean tapering during the intervention year was 83.9% in the tapering/discontinuation group and 50.0% in the maintenance group. Although in some participants, the dose was increased after tapering.

**Table 2:** Antipsychotic medication use in patients with first-episode schizophrenia at baseline, during the intervention year and at one-year follow-up.

	**Tapering/discontinuation group**	**Maintenance group**	**Total**
**Baseline**	**N** = **14**	***N*** = **15**	***N*** = **29**
Antipsychotic medication in mg haloperidol equivalents, mean / (range)	7.3/(2.7–11.3)	8.3/(2.4–16.0)	7.8/(2.4–16.0)
First- generation antipsychotics, count (column %)	0 (0)	0 (0)	0 (0)
Second-generation antipsychotics, count (column %)	14 (100)	15 (100)	29 (100)
Long-acting injectable antipsychotic medication, count (column %)	7 (50)	3 (20)	10 (35)
Oral antipsychotic medication, count (column %)	8 (57)	14 (93)	22 (76)
Antipsychotic medication polypharmacy, count (column %)	2 (14)	2 (13)	4 (14)
**Intervention**	*N* = 14	*N* = 15	*N* = 29
% tapering^a^, mean / range	83.9 / (25.0–100.0)	50.0 / (0.0–100.0)	66.4 / (0.0–100.00)
**1-year follow-up**	*N* = 12	*N* = 13	*N* = 25
Antipsychotic medication – yes, count (column %)	5 (41.7)	9 (69.2)	14 (56.0)
Antipsychotic medication in mg haloperidol equivalents for all (mean / range)	3.8 / (0–13.3)	5.7 / (0–16.0)	4.8 / (0–16.0)
First-generation antipsychotic medication, count (column %)	1 (8)	1 (8)	2 (8)
Second-generation antipsychotics, count (column %)	5 (42)	9 (69)	14 (56)
Long-acting injectable antipsychotic medication, count (column %)	2 (17)	2 (15)	4 (16)
Oral antipsychotic medication, count (column %)	3 (25)	8 (62)	11 (44)
Antipsychotic medication polypharmacy, count (column %)	1 (8)	2 (15)	3 (12)

a *Data from 29 included patients. Data from two patients was incomplete during intervention year because they moved*.

Participants and clinicians reported that the reasons for low adherence to the treatment arm in the maintenance group were primarily due to participants wanting to discontinue antipsychotics and, to a lesser extent unacceptable side effects, although this was not systematically investigated.

### Outcomes at 1-year follow-up

The primary, secondary and exploratory outcomes measured at 1-year follow-up and safety measures during the 1-year intervention are listed in Table 3. Overall, more participants in the discontinuation group, compared to the maintenance group, discontinued antipsychotic medication and remained in remission of psychotic symptoms.

**Table 3:** Primary, secondary and exploratory outcomes at one-year follow-up and safety measures during the 1-year intervention among patients with first-episode schizophrenia.

	**Tapering/discontinuation group (*****n*** = **12)**	**Maintenance group (*****n*** = **13)**	**Total (*****n*** = **25)**
**Primary outcome**	**Mean (SD)**	**Mean (SD)**	**Mean (SD)**
Remission of psychotic symptoms (SAPS≤ 2 in all global scores in minimum 3 months) and no antipsychotic medication in past 3 months^*^, count (column %)	5 (41.7%)	2 (15.4%)	7 (28.0%)
**Secondary outcomes**			
Remission of psychotic symptoms (SAPS ≤ 2 in all global scores in minimum 3 months) and antipsychotic medication > 0- and ≤ 1-mg haloperidol equivalents in past 3 months before, count (column %)	0	0	0
Psychotic symptoms (SAPS psychotic dimension)	1.5 (1.32)	0.9 (1.2)	1.2 (1.3)
Antipsychotic medication in mg haloperidol equivalents at 1-year follow-up	3.8 (5.0)	5.7 (5.1)	4.8 (5.0)
Side effects (UKU^§^)			
Psychic side effects^a^	11.8 (6.7)	4.0 (3.6)	7.0 (6.2)
Neurologic side effects^a^	2.0 (2.8)	1.1 (1.5)	1.5 (2.0)
Autonomic side effects	3.4 (3.4)	1.6 (1.9)	2.2 (2.6)
Other side effects^b^	3.3 (2.5)	1.3 (2.8)	2.1 (2.7)
Patient score of interference	1.8 (1.3)	1.0 (1.0)	1.3 (1.1)
Rater score of interference	1.4 (0.9)	1.1 (0.8)	1.2 (0.8)
Negative symptoms (SANS negative dimension)	1.5 (0.8)	1.8 (0.8)	1.6 (0.8)
Social function (GSDS score except parental role)	6.3 (2.7)	7.2 (2.2)	6.8 (2.5)
Functional remission 3 months (GSDS cut off), count (column %)	3 (25.0%)	1 (7.7%)	4 (16.0%)
Cognitive function (BACS)	−1.1 (1.9)	−2.7 (1.0)	−1.9 (1.7)
Client satisfaction (CSQ^c^)	28.8 (3.3)	28.8 (4.2)	28.8 (3.7)
**Exploratory outcomes**			
Social function (PSP total score)	55.0 (9.2)	60.2 (7.7)	57.7 (8.7)
Level of functioning (GAF-F)	54.1 (8.4)	58.2 (6.8)	56.2 (7.8)
	Count	Count	Count
	(column %)	(column %)	(column %)
Remission in past 3 months: remission of psychotic symptoms (SAPS ≤ 2 in all global scores), negative symptoms (SANS ≤ 2 in all global scores) and functional remission (GSDS ≤ 1 in all roles simultaneously)	3 (25.0%)	1 (7.7%)	4 (16.0%)
Remission in past 6 months: remission of psychotic symptoms (SAPS ≤ 2 in all global scores), negative symptoms (SANS ≤ 2 in all global scores) and functional remission (GSDS ≤ 1 in all roles simultaneously)	1 (8.3%)	1 (7.7%)	2 (8.0%)
Sexual dysfunction (CFSQ-14 cut off)^d^	5 (55.6%)	2 (16.7%)	7 (33.3%)
Use of drugs (timeline follow back)	2 (16.7%)	2 (15.4%)	4 (16.0%)
	Median (range)	Median (range)	Median (range)
Alcohol units per day (timeline follow back)	3.5 (0.0–25.0)	2.0 (0.0–30.0)	2.0 (0.0–30.0)
	Mean (SD)	Mean (SD)	Mean (SD)
Quality of life (WHO-5 Wellbeing Index)^a^	60.0 (27.9)	64.0 (13.6)	62.0 (21.6)
Self-belief of coping (GSES mean)^a^	3.0 (0.7)	2.8 (0.5)	2.9 (0.6)
Service user's experience of support (INSPIRE-S)	75.7 (22.8)	85.4 (10.6)	80.7 (17.9)
Service user's experience of relationship (INSPIRE-R)	91.7 (14.7)	95.9 (7.3)	93.9 (11.4)
Health related quality of life (EQ-5D-3L)	0.89 (0.12)	0.93 (0.07)	0.91 (0.10)
**Safety measures^#^**	**Tapering/discontinuation group** **(*n* = 14)**	**Maintenance group** **(*n* = 15)**	**Total** **(*n* = 29)**
	Count	Count	Count
	(column %)	(column %)	(column %)
Patients with relapse (SAPS phone)	2 (14.3%)	3 (20.0%)	5 (17.2%)
Patients with a Serious adverse event or reaction (SAE/SAR)	2 (14.3%)	3 (20.0%)	5 (17.2%)

* *Antipsychotic medication use for 3 months was available in all patients except in one patient, where data was based on one month*.

§ *UKU only rated if the patient took antipsychotic medication (Tapering/discontinuation group n = 5 and Maintenance group n = 9)*.

# *Safety measures are reported for all patients included in the study*.

a *Missing in one patient in the maintenance group*,

b *Missing in one patient in the tapering/discontinuation group and three patients in the maintenance group*,

c *Missing in one patient in the tapering/discontinuation group and one patient in the maintenance group*.

d *Missing in three patients in the tapering/discontinuation group and one patient in the maintenance group*.

*BACS, Brief Assessment of Cognition in Schizophrenia; CSFQ-14, Changes in Sexual Functioning Questionnaire; CSQ, Client Satisfaction Questionnaire; EQ-5D-3L, European Quality of Life – 5 Dimensions; GAF-F, Global Assessment of Function; GSDS, Groningen Social Disabilities Schedule; GSES, General Self Efficacy; PSP, Personal and Social Performance Scale; SANS, Schedule for Assessment of Negative Symptoms; SAPS, Scale for the Assessment of Positive Symptoms; UKU, Udvalg for Kliniske Undersøgelser*.

Regarding safety, relapse occurred in two participants in the tapering/discontinuation group and three in the maintenance group. Two participants in the tapering/discontinuation group experienced serious adverse events i.e., admission to psychiatric hospital or having a suicide attempt. In the maintenance group, serious adverse events occurred in three participants i.e., admission to psychiatric hospital due to either suicidal ideations or psychotic symptoms or becoming pregnant. No deaths or other Suspected Unexpected Serious Adverse Reaction (SUSAR) occurred during the study.

## Discussion

In this randomized clinical trial, we studied whether patients with first-episode schizophrenia could sustain remission of psychotic symptoms after tapered discontinuation of antipsychotic medication compared to maintaining antipsychotic medication during a 1-year intervention in early intervention services. However, the recruitment was insufficient and adherence challenging. The trial terminated early, and due to the small sample size, no outcomes were reported with statistical analyses. Comparison between groups was problematic because of the limited number of participants and unexpected similar interventions. Therefore, the primary and other outcomes are left unanswered making it difficult to compare results with the existing literature. Therefore, no conclusion can be inferred with regard to the original aim. Although the sample was too small, this report is important to create transparency and to discuss trial designs and clinical implications to obtain better trials and more knowledge of antipsychotic maintenance treatment of patients with first-episode schizophrenia.

### Recruitment issues

The TAILOR trial failed to recruit enough participants and thus ended prematurely. In a review of 172 RCTs discontinued due to poor recruitment, Briel et al. identified 28 distinct reasons for recruitment failure (20). Most of the reasons are not relevant to our trial, but we believe some of the identified reasons played a part in why we failed in our recruitment:

- Overestimated prevalence: Our recruitment strategy was based on calculations from previous studies of patients treated in the OPUS teams fulfilling our inclusion criteria. However, the calculations did not fit the clinical reality, given that many referred participants did not fulfill the inclusion criteria. We tried to address this by expanding the timeframe, but this did not affect recruitment. Furthermore, as the tapering intervention was readily available outside the trial, patients did not necessarily have an incitement to participate.
- Concerns regarding side effects or potential diagnosis: Among clinicians, a concern was that continuing antipsychotic medication would cause unnecessary side effects and being against the patient's wish.
- Lack of engagement (e.g., recruiters were not part of the study team): We experienced that the clinicians were interested in the question of feasibility and safety of discontinuation, but as they were not part of the research team, this might have influenced their engagement in the study.
- General mistrust in research: We did not experience a general mistrust in research, *per se*, but participants were reluctant to let randomization decide whether to continue or discontinue antipsychotic medication, even if the evidence supporting either option is scarce.
- Prejudice against effectiveness of trial interventions: To some degree, the recruitment may have been influenced by the clinicians' concern that patients entering the trial would experience a relapse when discontinuing antipsychotics.

The reasons for preferring continued or discontinued treatment with antipsychotics in a randomized clinical trial resemble reasons reported in clinical settings, such as medication efficacy, side effects, personal beliefs, and the influence of social relationships (21). Therefore, enrolment in a randomized trial could not override these beliefs or the influence of social relationships on treatment, which should be encountered in future studies. A previous study showed similar concerns among English clinicians but less apprehension regarding discontinuation of antipsychotics compared to guidelines (22). Patients included in this trial were highly selected due to the narrow inclusion criteria of the study and the necessity for patients and clinicians to accept the risk of tapering. This acceptance may have been facilitated through patient's own belief of coping or the clinician's belief of predictors existing for good outcome. Previously reported predictors for relapse following discontinuation in first-episode psychosis are e.g., male sex, unemployment, prior psychiatric hospital admission, poor premorbid function, schizophrenia diagnosis, concomitant medication, and more negative symptoms (23). However, factors such as DUP and dose of antipsychotics were not predictive. The mean dose of antipsychotic medication at inclusion was 7.8 mg haloperidol equivalents (SD 3.3), which was higher than expected but possibly because no reduction to a minimal effective dose in the maintenance phase was yet achieved. In comparison, 4 mg haloperidol equivalents has previously been defined as the minimal effective dose (18).

### Difficulties in adherence to maintenance treatment

Regarding adherence, most participants in the maintenance group tapered or discontinued their antipsychotic medication. The low adherence to maintenance treatment reflects problems with maintaining the dose for a year, which is also reported in naturalistic studies (24, 25). Many patients with first-episode schizophrenia and remitted psychotic symptoms wish to taper their antipsychotic medication and will do so regardless of being in a clinical trial. Whether a discontinuation trial led patients wishing to discontinue their antipsychotic medication is a hypothetical possibility. Therefore, only clinicians were exposed to advertisements for the trial.

In efficacy trials, the rigidity of interventions collides with the concept of shared decision-making and flexible treatment and is not easily transferable to real-world settings. The present study had a more pragmatic design to test the effectiveness of slow, gradual, and guided tapering as it was considered to improve recruitment, avoid extensive dropouts, and make it easier to transfer results to clinical settings, However, in hindsight monitoring interventions in the trial should have been more intense to ensure adherence to protocol and enable accurate intention-to-treat analyses.

### Alternative designs

The question remains whether tapering/discontinuation in patients with first-episode schizophrenia may have advantages compared to maintenance treatment. As discussed above, setting up a trial has several challenges which future researchers may overcome. We believe a new randomized controlled trial on several parameters should resemble an efficacy trial (26). Researchers should handle patients' medical care, including the intervention of tapering and using a placebo for antipsychotic medication to ensure adherence to the treatment protocol and prohibit the participants and clinicians from breaking protocol. However, the use of placebo may impose ethical considerations of safety because the interpretation of side effects, effects, and early signs of deterioration and relapse cannot be referred to antipsychotics, and treatment cannot be adjusted accordingly. Furthermore, the recruitment area should be broadened and inclusion criteria wider e.g., by including all schizophrenia spectrum disorders. We recommend that future trials design and conduct pilot studies in close collaboration with clinicians and patients to ensure recruitment and ascertain feasibility. Furthermore, the engagement of clinicians and integration of research into clinical settings may also increase recruitment and ease monitoring.

Although randomized controlled trials are regarded as the gold standard for evaluating clinical interventions, the implications are that similar trials will encounter similar recruitment and adherence problems. Studies with different designs (27) may overcome obstacles to recruitment and adherence. The first suggestion is to change the setting to “tapering clinics” where gradual discontinuation of antipsychotics is offered together with close follow-ups, assertive community treatment, and involvement of relatives. The second suggestion is to study gradual discontinuation as an intervention in a single-arm clinical trial (i.e., with no control group). The third suggestion is to examine discontinuation in a non-interventional naturalistic design with a clinical cohort of patients with first-episode schizophrenia in remission of psychotic symptoms. The patients may be selected based on the existing knowledge on predictors of good outcomes when discontinuing antipsychotics, thus increasing safety for study participants. The fourth suggestion is to use observational data from register-based population studies and perform causal inference analyses to adjust for potential confounders and bias. The designs of suggestions three and four allow shared decision-making due to no interventions and hence the opportunity to include patient preferences and values. The final suggestion is to develop n-of-1 trials to personalize treatments, although use of the this method has been limited and characterized by a high risk of bias (28).

Ethical considerations of risks are essential when conducting discontinuation trials, as are reflections on risks of long-term treatment with antipsychotic medication. Future trials of discontinuing antipsychotics should still focus on patient-oriented goals such as functional remission, quality of life, and personal recovery, as there is a knowledge gap regarding these outcomes (8). The TAILOR trial reflected feasibility and implicated planning a new clinical study, TAILOR 2, with a prospective naturalistic follow-up design. Results presented in this article may be combined with other ongoing comparable discontinuation trials (29–31), so participants have not been exposed to unnecessary burdens and risks. Furthermore, the aggregation of results may contribute knowledge and evidence of whether or when it is favorable for patients to discontinue antipsychotic medication.

## Conclusion

Due to insufficient recruitment this study does not provide a conclusion on whether tapering antipsychotic medication is followed by unfavorable outcomes such as the risk of relapse or advantages. Recruitment and adherence to randomization of maintenance treatment encountered severe obstacles. Thus, future studies should explore the pros and cons of continuing vs. discontinuing antipsychotic medication in patients with first-episode schizophrenia and consider which designs are feasible and relevant in this quest.

## Data availability statement

The datasets presented in this article are not readily available because of restrictions on data sharing determined by the Danish Data Protection Agency. Requests to access the datasets should be directed to anne.emilie.stuerup@regionh.dk.

## Ethics statement

This study involved human participants and the study was reviewed and approved by Regional Research Ethical Committee in the Capital Region of Denmark (no 53322). The patients/participants provided their written informed consent to participate in this study.

## Author contributions

MN was the trial sponsor, directed the planning of the study design, took part in the interpretation of data and the decision to submit the report for publication, had the authority over the project, and obtained funding. MN and OM were primary initiators of the project and both were principal investigators. MN, OM, LE, BE, and CH consisted the steering committee, decision-making on project design, alterations, cooperation, budget, and data handling. MN, OM, LE, BE, CH, AS, SD, HJ, and MB collaborately did the development of the protocol. MN, OM, and DV made recruitment possible in their respective regions of Denmark. SD, HJ, MB, NA, HS, and AS recruited, interviewed, and tested patients at baseline and 1-year follow-up. AS wrote the first draft of the manuscript, data management, and extraction in SPSS. MN, OM, LE, BE, CH, DV, SD, HJ, MB, NA, and HS have done critical revision. All authors approved the final version.

## Funding

The TAILOR trial was funded by TrygFonden in Denmark (Grant Number 109436) (MN), The Capital Region of Denmark (MN) and The Medication Fund of the Regions of Denmark (Grant Number 15/1716) (MN). The Central Denmark Region and The Capital Region of Denmark supported the study indirectly by allowing the staff to follow the tapering manual but had no other influence on the study. The funders had no role in the study design, data collection, management, analysis and interpretation of data, writing the manuscript or the decision to submit for publication.

## Conflict of interest

BE is part of the Advisory Board of Eli Lilly Denmark A/S, Janssen-Cilag, Lundbeck Pharma A/S, and Takeda Pharmaceutical Company Ltd; and has received lecture fees from Bristol-Myers Squibb, Otsuka Pharma Scandinavia AB, Eli Lilly Company, Boehringer Ingelheim Denmark A/S, and Lundbeck Pharma A/S. DV has received speaking fees from Lundbeck. The remaining authors declare that the research was conducted in the absence of any commercial or financial relationships that could be construed as a potential conflict of interest.

## Publisher's note

All claims expressed in this article are solely those of the authors and do not necessarily represent those of their affiliated organizations, or those of the publisher, the editors and the reviewers. Any product that may be evaluated in this article, or claim that may be made by its manufacturer, is not guaranteed or endorsed by the publisher.
